# Consequences of CRISPR‐Cas9‐Mediated Stromelysin‐1 Knockout in Pancreatic Islet Microvascular Endothelial Cells

**DOI:** 10.1111/jcmm.71098

**Published:** 2026-03-20

**Authors:** Bing Wang, Weiqi Liu, Yuan Li, Qin Ouyang, Yingyu Wang, Xiang Xu, Bingwei Li, Ruijuan Xiu, Xu Zhang, Mingming Liu

**Affiliations:** ^1^ Institute of Microcirculation, Chinese Academy of Medical Sciences & Peking Union Medical College Beijing China; ^2^ International Center of Microvascular Medicine, Chinese Academy of Medical Sciences Beijing China; ^3^ Diabetes Research Center, Chinese Academy of Medical Sciences Beijing China; ^4^ Department of Pathology, Wangjing Hospital, China Academy of Chinese Medical Science Beijing China; ^5^ Laboratory of Electron Microscopy, Ultrastructural Pathology Center Peking University First Hospital Beijing China

**Keywords:** endothelial dysfunction, glucotoxicity, islet microvascular endothelial cells, secretome, stromelysin‐1

## Abstract

The integrity of the pancreatic islet microvasculature is critical for endocrine function, yet it is progressively compromised by glucotoxicity in diabetes. While matrix metalloproteinases are implicated, the role of stromelysin‐1 as a potential upstream driver of endothelial dysfunction remains poorly defined. The aim of our study was to elucidate the role of stromelysin‐1 in mediating glucotoxic injury to islet microvascular endothelial cells (IMECs). To this end, we employed a CRISPR/Cas9‐mediated knockout of stromelysin‐1 in IMECs. Cellular functions, including proliferation, migration, and angiogenesis, were assessed using IncuCyte ZOOM live‐cell imaging, while endothelial barrier integrity was quantified via a 40 kDa dextran flux assay. Additionally, the secretome was profiled using a cytokine antibody array. We found that genetic ablation of stromelysin‐1 conferred protection against glucotoxicity. Stromelysin‐1 KO IMECs exhibited significantly enhanced proliferation, migration, and angiogenic capacity compared to wild‐type controls. Furthermore, stromelysin‐1 deficiency restored endothelial monolayer integrity by attenuating high‐glucose‐induced hyperpermeability. These functional improvements were linked to a remodelling of the secretome, characterised by decreased secretion of the pro‐degradative MMP‐2 and increased secretion of the anti‐inflammatory cytokine IL‐10 and the endogenous inhibitor TIMP‐2. Overall, our findings establish stromelysin‐1 as a crucial mediator of glucotoxic injury in islet microvascular endothelial cells.

## 1 Introduction

Diabetes mellitus is a heterogeneous, complex metabolic disorder characterised by elevated blood glucose concentrations secondary to either resistance to the action of insulin or insufficient insulin secretion. Generally, the emphasis on macro‐circulation ignores the contribution of the microcirculation to diabetes mellitus [1]. As a highly vascularized tissue, the integrated pancreatic microcirculation is responsible for the exchanges of oxygen and nutrients in both endocrine and exocrine functions and the regulation of glucose metabolic homeostasis through insulin transportation [2, 3]. Recently, accumulating experimental evidence indicates that microcirculatory disturbance, as both a cause and a consequence, is involved in the onset and the development of diabetes and its complications [4].

The pancreatic islet microcirculation is a highly vascularized capillary network lined by a distinctive fenestrated endothelium with a density of fenestrae in islet microvascular endothelial cells (IMECs) [5, 6]. For decades, the relationship between hyperglycemia and insulin resistance in the pathogenesis of microvascular dysfunction remained a matter of debate. Historically, diabetic microangiopathy was largely attributed to the direct effects of glucotoxicity, whereas insulin resistance was predominantly associated with macrovascular complications. However, current evidence indicates that hyperglycemia and localized endothelial insulin resistance are mechanistically interconnected and act synergistically to disrupt microvascular barrier integrity and promote endothelial dysfunction. However, functional and morphological alterations of microvascular endothelial cells (ECs) would lead to microcirculatory disturbances, thereby providing a causal role in the pathogenesis of diabetic vascular disease. The structural and functional stability of IMECs is a prerequisite for maintaining homeostasis of the pancreatic islet microcirculation. Several studies, including ours, have proposed that deteriorated IMECs progressively impair the pancreatic islet microenvironment, creating a favourable pathological niche. The functional integrity of IMECs is tightly governed by endogenous regulators, and its disruption is frequently driven by the aberrant activation of matrix metalloproteinases (MMPs) [7].

MMPs are a family of zinc‐dependent proteolytic enzymes that degrade various components of the ECM in both physiological and pathological processes to facilitate the progression of cell migration and proliferation, extracellular matrix remodelling, and angiogenesis. Abnormal microhemodynamic force, which occurred in the diabetic pancreas established by our group, might activate MMP‐2 and MMP‐9, leading to the unchecked cleavage of extracellular domains of functional membrane proteins (such as VEGFR‐2) [8, 9]. Additionally, we have previously reported that the stromelysin‐1 (matrix metalloproteinase 3) may have a putative role in glucotoxicity‐exposed IMECs dysfunction through the analysis of gene expression profile [10]. However, the significance of stromelysin‐1 in IMECs was still unknown. To interrogate the mechanisms underlying glucotoxicity‐induced IMEC dysfunction and to benchmark the protective efficacy of stromelysin‐1 ablation, we employed three different rescue agents: insulin, l‐arginine (LA), and β‐mercaptoethanol (βME). In the islet microenvironment, IMECs are continuously exposed to high local insulin concentrations, which normally provide vasoprotective signalling via eNOS activation [11, 12]; however, glucotoxicity impairs the signalling pathway and diminishes its protective capacity. l‐Arginine, the obligate substrate for eNOS, was utilised to metabolically bypass oxidative eNOS uncoupling and restore nitric oxide bioavailability [13, 14]. While βME was selected as a potent chemical antioxidant to directly scavenge reactive oxygen species, which are known upstream drivers of aberrant MMP activation [15, 16]. Together, these agents serve as physiological, enzymatic, and chemical positive controls, respectively, allowing us to investigate the intersection between oxidative stress, endothelial signalling, and stromelysin‐1‐mediated injury.

## 2 Materials and Methods

### 2.1 Cell Line and Treatment Conditions

A pancreatic islet microvascular endothelial cell line (MS1; ATCC, Manassas, VA, USA) was employed in the present study, and its culture was performed in specifically formulated media. The basal medium consisted of Dulbecco's Modified Eagle Medium (DMEM) containing 5.6 mM glucose, supplemented with 10% fetal bovine serum (FBS), 2% HEPES (Gibco, Carlsbad, CA, USA), and a 1% penicillin–streptomycin solution (containing 100 U/mL penicillin and 100 μg/mL streptomycin) to maintain cell viability and prevent microbial contamination. The high‐glucose (HG) treatment medium was formulated using low‐glucose DMEM, with D‐glucose subsequently added to reach a final concentration of 35 mM to establish the high‐glucose experimental model. Additive‐supplemented treatment media were prepared by further modifying the high‐glucose treatment medium with a single reagent at a final concentration of 10^−8^ M insulin, 0.5 mM l‐arginine, or 100 μM β‐mercaptoethanol (βME).

All cell cultures were maintained in a humidified incubator at 37°C with 5% CO₂ to mimic physiological conditions. When reaching confluence, both wild‐type and stromelysin‐1 KO MS1 cells were divided into a 5.6 mM glucose‐treated group (Control), a 35 mM glucose‐treated group (HG), a 35 mM glucose plus 10^−8^ M insulin‐treated group (HG + I), a 35 mM glucose plus 0.5 mM l‐arginine‐treated group (HG + LA), and a 35 mM glucose plus 100 μM βME‐treated group (HG + βME).

### 2.2 Generation of Stromelysin‐1 CRISPR/Cas9 KO in MS1 Cells and Verification

A homozygous knockout (KO) of stromelysin‐1 in MS1 was achieved using a lentiviral transduction‐based CRISPR/Cas9 technique. Briefly, the full‐length stromelysin‐1 was cloned into pLentiCRISPRv2 vectors. The sequences of the primers used to clone stromelysin‐1 were as follows: forward: 5′‐GTTGGGCTTAAGAAGGTGGAC‐3′; and reverse: 5′‐TTTAATAAACGACACACA ATCTTTATTA‐3′. The sgRNA for stromelysin‐1 was designed using the open‐source platform (https://zlab.bio/guide‐design‐resources) as follows: 5′‐ACTTTGACGATGATGAACGA‐3′, and this sgRNA was subsequently cloned into lentiCRISPR v2.0. Subsequently, cells were transfected with lentiCRISPR v2.0‐stromelysin‐1 along with packaging plasmid (pMD2.G) to generate the lentiviruses. At 48–72 h post‐transfection, the cell supernatants containing lentiviruses were collected. Then, MS1 cells were infected with lentiviruses in the presence of 1 μg/mL puromycin (Meilunbio, Dalian, China) to construct a stable‐transfected stromelysin‐1 KO cell line.

The successful knockout of stromelysin‐1 in the stable cell line was confirmed by Western blot analysis. Briefly, total protein was extracted from cells, separated by SDS–PAGE, and transferred onto PVDF membranes. After blocking with 5% non‐fat milk in TBST, membranes were incubated overnight at 4°C with a rabbit anti‐stromelysin‐1 polyclonal primary antibody (ab137659, Abcam, Cambridge, UK) at a dilution of 1:1000. Following washing, membranes were incubated with a horseradish peroxidase (HRP)‐conjugated goat anti‐rabbit IgG secondary antibody (ab97051, Abcam, Cambridge, UK) at a dilution of 1:5000 for 1 h at room temperature. Protein bands were visualised using enhanced chemiluminescence (34,577, Thermo Fisher Scientific, Waltham, MA, USA).

### 2.3 Cell Proliferation Assay

The cell proliferation assay was performed using the IncuCyte ZOOM live cell imaging system (Essen BioScience, MI, USA). After a total of 100 μL of both WT and stromelysin‐1 KO cell suspension was incubated in a 96‐well plate at a density of 4 × 10^3^ cells per well, cells were then grown overnight to form a spatially uniform monolayer. Subsequently, 100 μL of treatment medium was added to each well, and the plate was placed into the IncuCyte ZOOM system. Images of the collective cell spreading were recorded every 12 h for a total duration of 72 h.

### 2.4 Cell Migration Assay

WT and stromeysin‐1 KO MS1 were maintained in a 96‐well ImageLock plate (Essen BioScience) at 2 × 10^4^ cells/well until 100% confluent monolayer growth was obtained. As previously described, the monolayer was scratched by WoundMaker (Essen BioScience). Subsequently, the medium was aspirated, and the wells were washed twice with fresh medium to remove any unattached cells and to smooth the edges of the scratch area. Once the treatment media mentioned above were added, the plate was placed into the IncuCyte ZOOM system, and imaging was recorded every 24 h for a total duration of 48 h. The quantitative analysis was performed by the integrated analysis algorithm of the IncuCyte ZOOM software; the changes in both wound width and wound confluence were calculated by the standard IncuCyte masking procedure.

### 2.5 Matrigel Tube Formation Assay

The tube formation assay was performed as described previously [17]. Briefly, a 96‐well plate was coated with Matrigel (BD Biosciences) and incubated for 30 min at 37°C to achieve polymerisation of the gel. Subsequently, WT and stromelysin‐1 KO MS1 cells were suspended and seeded into the plates at a density of 2 × 10^4^ cells/well. Following a 24 h incubation with the treatment medium in the IncuCyte ZOOM system, fields of triplicate wells were automatically photographed every 3 h and quantified using WimTube Release 4.0 via the WIMASIS Image Analysis System (WIMASIS, https://www.wimasis.com/en/products/WimTube). For quantification, a loop was defined as a closed polygonal area enclosed by interconnected endothelial tubular segments. The total number of intact loops per field of view was automatically recognised and counted by the WimTube software to provide a quantitative measure of network formation.

### 2.6 Permeability Analysis

The monolayer permeability of MS1 was determined by the flux of fluorescein isothiocyanate (FITC)‐labelled dextran (40‐kDa molecular weight) through endothelial cell monolayers on a transwell system. WT and stromelysin‐1 KO (5 × 10^4^ cells/well) were seeded in the upper chamber of 3 mm pore polyethylene membrane inserts in 24‐well plates (Corning, Tewksbury, MA, USA). The cells were subsequently starved for 4 h and exposed to the treatment medium for 48 h. FITC‐labelled dextran (Sigma‐Aldrich, St. Louis, MO, USA) was then added to the upper well. 100 μL aliquots were collected from the lower layer at 0, 5, 15, 30, 60, and 90 min, respectively. After transferring to a 96‐well plate, the fluorescence of FITC leaked to the lower chamber was measured with a fluorescence multiple plate reader (Bio‐Tek, Winooski, VT, USA) at 490 nm/510 nm. The fluorescence at each time point was calculated and normalised against the fluorescence value at baseline.

### 2.7 Secretome Profiling by Cytokine Array

The preliminary multianalyte screening for the differentially expressed cytokines of supernatants between stromelysin‐1 KO and WT MS1 was performed using 96‐cytokines array membranes (ab193659; Abcam, Cambridge, UK) according to the manufacturer's protocol. The chemiluminescent signals were recorded by the Tanon‐4200 imaging system (Tanon, Shanghai, China). The intensity of signals was quantified with ImageJ 5.0 (http://rsb.info.nih.gov/ijlecular, National Institute of Health, Bethesda, MD, USA). For each analyte, the signal was corrected for background signal and normalised to the signal of the membrane reference spots.

### 2.8 Statistical Analysis

Statistical analysis was performed using SPSS version 19.0 (SPSS Inc., Chicago, IL, USA). All the values were represented as the mean ± standard error of the mean (SEM) of at least three independent experiments. For the comparisons between two groups of values, the statistical analysis of the results was performed by the Student's *t*‐test. *p* values < 0.05 were statistically significant.

## 3 Results

### 3.1 Stromelysin‐1 KO Increased Proliferation Capacity of IMECs

**FIGURE 1:**
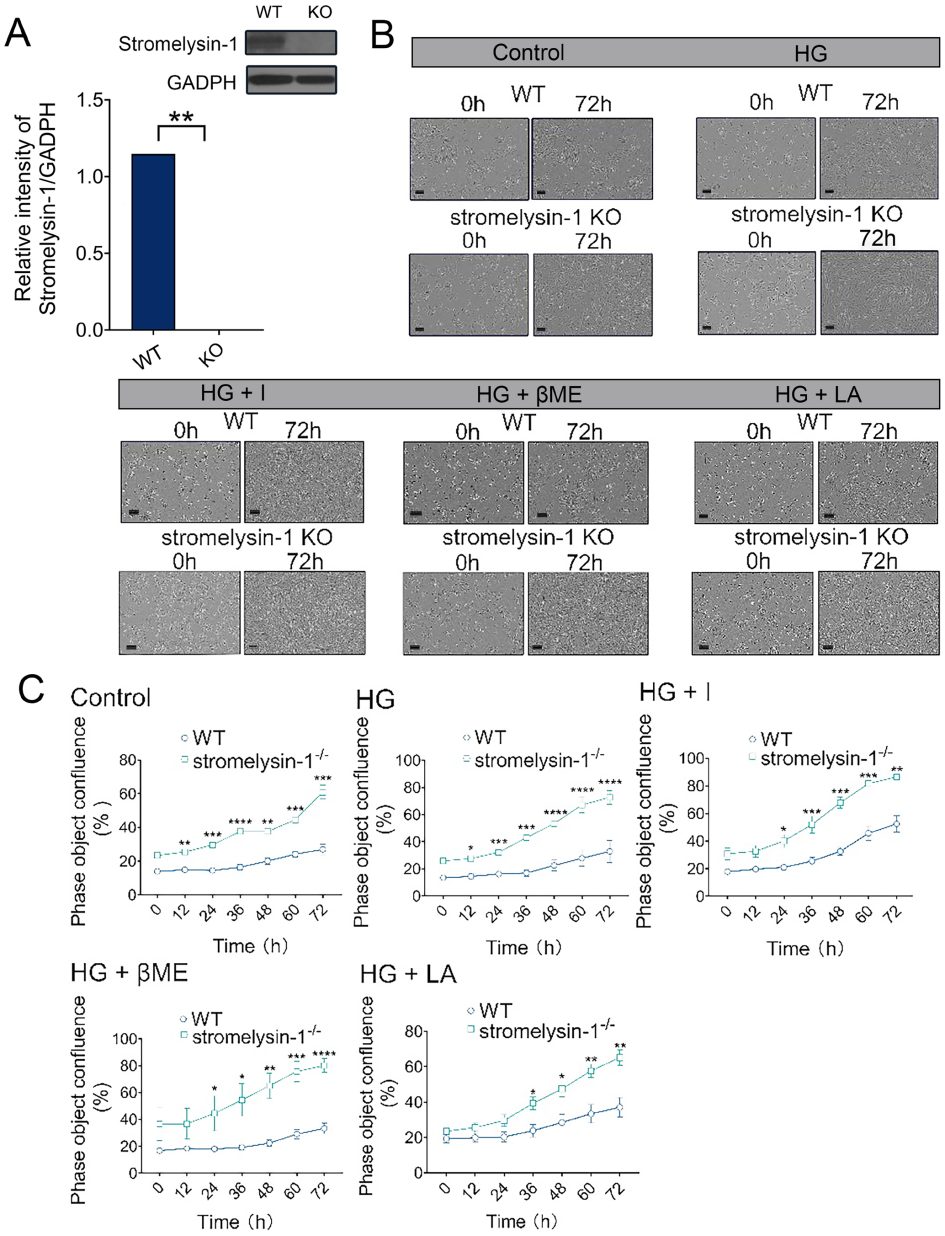
Stromelysin‐1 KO promotes IMEC proliferation and confers resistance to glucotoxicity. (A) Validation of stromelysin‐1 knockout. Representative immunoblot and corresponding densitometric quantification showing the depletion of stromelysin‐1 protein in knockout (KO) IMECs compared to wild‐type (WT) controls. (B) Quantification of IMEC proliferation, assessed by phase object confluence over 72 h. WT and stromelysin‐1 KO cells were cultured under normal glucose (Control, 5.6 mM), high glucose (HG, 35 mM), and HG supplemented with 10^−8^ M insulin (HG + I), 0.5 mM l‐arginine (HG + LA), or 100 μM β‐mercaptoethanol (HG + βME). Representative images at 72 h are shown. Scale bar = 100 μm. (C) Quantitative analysis of cell proliferation measured as the percentage of phase object confluence. WT, wild‐type. KO, knockout. IMECs, islet microvascular endothelial cells. Data are presented as mean ± SEM (*n* = 3). *p < 0.05, **p < 0.01, ***p < 0.001, ****p < 0.0001 versus the corresponding WT group under the same treatment conditions.

We generated a stromelysin‐1 KO MS1 using the CRISPR/Cas9 genome editing system. Stromelysin‐1 KO was first determined visually through identification of flow cytometry after puromycin selection with transfection efficiency > 85% (data not shown). To confirm stromelysin‐1 KO, we measured stromelysin‐1 expression via Western blotting. Stromelysin‐1 protein expression in stromelysin‐1 KO MS1 was also significantly reduced via western blot (representative blot in Figure 1A). To assess the role of stromelysin‐1 on the glucotoxicity exposed IMECs, we first measured the proliferation capacity in both WT and stromelysin‐1 KO IMECs in the treatment medium. Figure 1B showed the growth and proliferation of WT and stromelysin‐1 KO cells over a total 72‐h exposure to treatment medium. To quantify the difference, we performed the image analysis with the IncuCyte Zoom system and found an increase in the phase object confluence of the stromelysin‐1 KO cells when compared with WT cells under normal glucose conditions. Stromelysin‐1 KO resulted in significantly enhanced proliferation of IMECs relative to WT after 48 h of high glucose stimulation (Figure 1C). In addition, stromelysin‐1 KO produced synergistic development of IMECs proliferation by insulin, LA, and βME treatment. Together, these data indicate that stromelysin‐1 affects the proliferation of IMECs to glucotoxicity.

### 3.2 Stromelysin‐1 KO Increased Migration Capacity of IMECs

**FIGURE 2:**
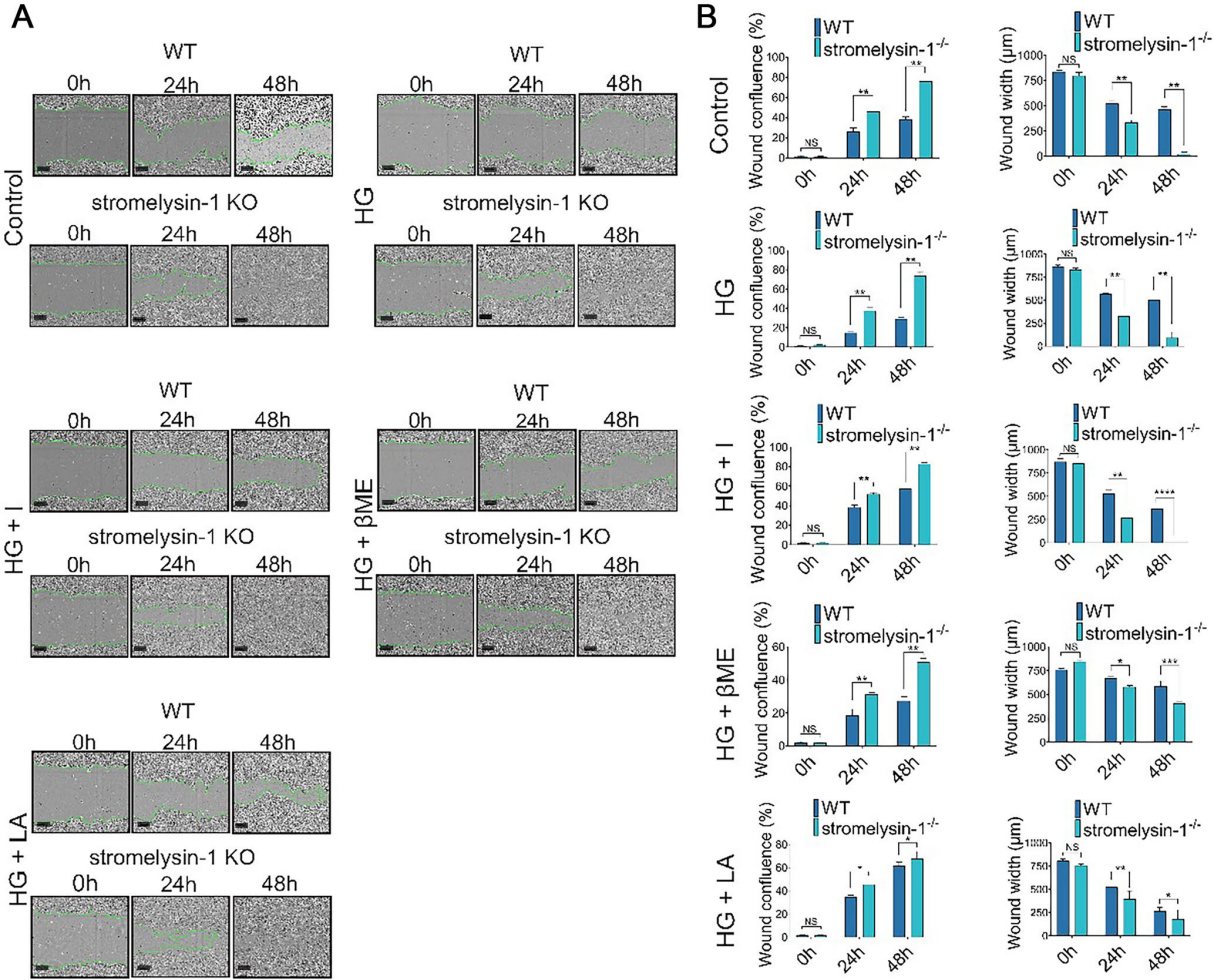
Stromelysin‐1 KO enhances the migratory capacity of IMECs in a wound healing assay. (A) Representative images of a scratch wound healing assay at 0, 24, and 48 h. WT and stromelysin‐1 KO IMECs were subjected to Control (5.6 mM glucose), HG (35 mM glucose), HG + I (insulin, 10^−8^ M), HG + LA (l‐arginine, 0.5 mM), and HG + βME (β‐mercaptoethanol, 100 μM), respectively. Scale bar = 100 μm. (B) Quantification of wound closure, presented as relative wound confluence at 24 and 48 h. WT, wild‐type. KO, knockout. IMECs, islet microvascular endothelial cells. Data are presented as mean ± SEM from three independent experiments. *p < 0.05, **p < 0.01, ***p＜0.001, ****p < 0.0001 compared with the corresponding WT group.

In addition to proliferation, increased endothelial cell migration is also required for vessel growth [18]. Subsequently, we further determine whether stromelysin‐1 KO functionally impacted the phenotypical migration of IMECs using the wound healing assay. As shown in Figure 2A, the deletion of stromelysin‐1 increased IMECs migration in response to the treatment medium. In stromelysin‐1 KO control groups, the wound confluence was significantly higher at 24 h (46.46% ± 14.98% vs. 26.27% ± 6.49%, respectively) and 48 h (76.49% ± 25.30% vs. 38.63% ± 32.62%, respectively; Figure 2B). Similarly, in high glucose‐exposed IMECs, the wound confluence was significantly increased at 24 h (14.91% ± 1.47% for WT vs. 37.78% ± 6.10% for stromelysin‐1 KO) and 48 h (29.27% ± 2.05% for WT vs. 73.58% ± 7.45% for stromelysin‐1 KO; Figure 2B). Given the findings that oxidative stress may be involved in the IMECs dysfunction derived from glucose toxicity, the wound healing recovery was evaluated in the presence of insulin, LA and βME. Quantification of wound width and confluence further confirmed that stromelysin‐1 KO produces synergistic induction of migration ability after treatment with insulin, βME and LA, respectively, indicating that the migration capability of glucotoxicity‐exposed IMECs was reversed by stromelysin‐1 KO.

### 3.3 Stromelysin‐1 KO Increased Angiogenetic Capability

**FIGURE 3:**
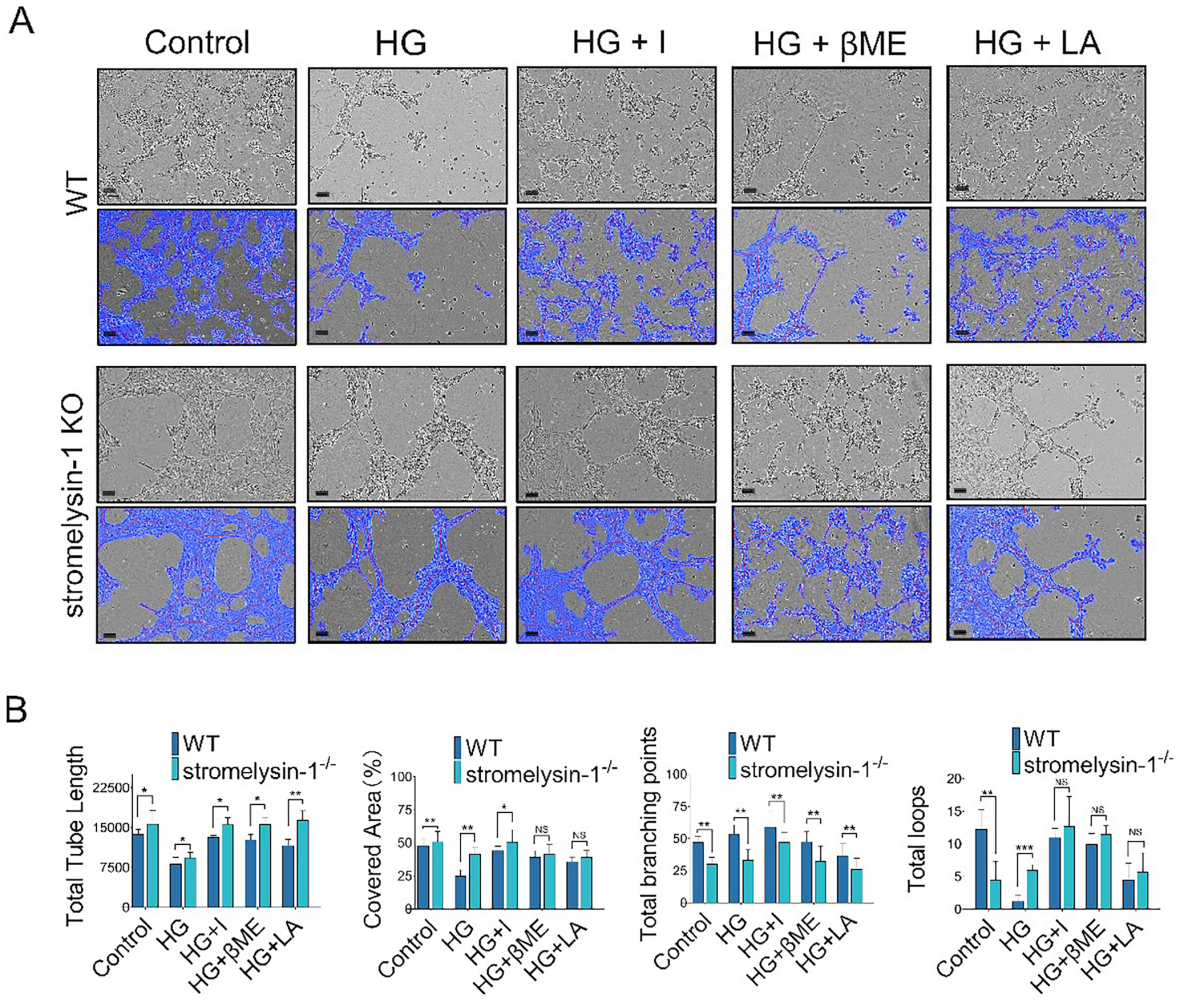
Stromelysin‐1 KO promotes in vitro angiogenesis of IMECs. (A) Representative images of capillary‐like network formation by WT and stromelysin‐1 KO IMECs cultured on Matrigel under 5.6 mM glucose (Control), 35 mM glucose (HG), 35 mM glucose plus 10^−8^ M insulin (HG + I), 35 mM glucose plus 0.5 mM l‐arginine (HG + LA) and 35 mM glucose plus 100 μM β‐mercaptoethanol (HG + βME) for 24 h (upper panel). Scale bar = 100 μm. Tubes and nets were automatically selected and marked in blue using Wimasis Image Analysis System (lower panel). Blue area represents the tubular structure. Red lines represent tubes. White points indicate branching points. (B) Quantification of tube formation capacity between WT and stromelysin‐1 KO. Data are expressed as mean ± SEM, *n* = 3. *p < 0.05, **p < 0.01, ***p < 0.001 compared with corresponding WT control group.

As both EC migration and proliferation are critical for angiogenesis, the effect of stromelysin‐1 in the IMECs was assessed by examining their ability to change morphology and form capillaries when cultured on Matrigel, which is a process mimicking sprouting and tube formation during angiogenesis in vitro. We compared them with WT cells in the treatment medium using the IncuCyte live‐content imaging system and quantified multiple metrics, including tube length, tube‐covered area and total loops. Figure 3A shows that stromelysin‐1 KO IMECs exhibited significantly increased tubule formation in the treatment medium. Quantitative analysis of multiple metrics indicates that the covered area (%), total tube length and the total loops in the stromelysin‐1 KO group were dramatically 1.6‐, 1.1‐ and 4.8‐fold higher than those in WT IMECs under high glucose conditions (Figure 3B). Furthermore, in the presence of insulin, LA and βME, stromelysin‐1 KO promoted the tube formation capability synergistically, suggesting that stromelysin‐1 negatively regulates the angiogenic capability of IMECs under high glycemia conditions.

### 3.4 Stromelysin‐1 KO Protected Against Glucotoxicity‐Induced Reduced Monolayer Permeability

**FIGURE 4:**
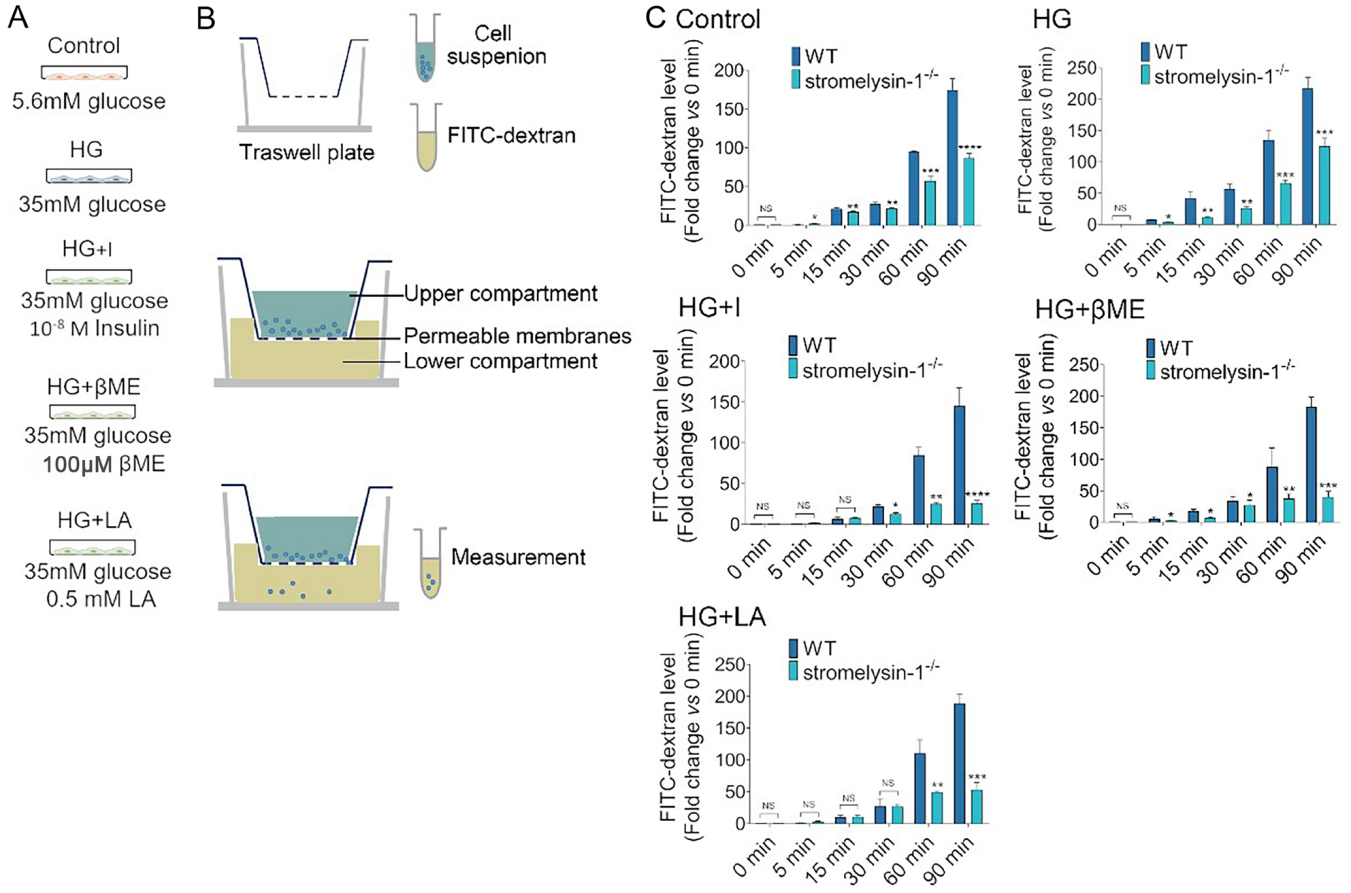
Stromelysin‐1 KO restores endothelial barrier integrity and attenuates glucotoxicity‐induced hyperpermeability. (A) Experimental groups and treatment conditions. (B) Schematic of the transwell‐based permeability assay. Confluent IMEC monolayers were exposed to the treatment media for 24 h, and the passage of 2 mg/mL FITC‐dextran from the upper to the lower chamber was measured over time. (C) Quantification of FITC‐dextran flux across IMEC monolayers over 90 min. Data show fluorescence intensity in the lower chamber, normalised to the baseline at *t* = 0. WT, wild‐type. KO, knockout. IMECs, islet microvascular endothelial cells. Control (5.6 mM glucose), HG (35 mM glucose), HG + I (insulin, 10^−8^ M), HG + LA (l‐arginine, 0.5 mM), and HG + βME (100 μM). Data are expressed as mean ± SEM, *n* = 3. *p < 0.05, **p < 0.01, ***p < 0.001, ****p < 0.0001, compared with corresponding WT group.

To confirm the functional injury of IMECs exposed to high glucose and verify whether the permeability changes were occurring by stromelysin‐1 KO, followed by FITC‐dextran added into the upper chamber of the Transwell system, the degree of FITC‐dextran permeability was then determined by measuring FITC‐dextran in the lower chambers using fluorometry (Figure 4A,B). Consistent with previous reports, we found that WT cells under high glucose conditions exhibited progressively higher FITC‐dextran levels in the lower chamber. Nevertheless, fluorescent levels at 60 and 90 min time‐points were diminished in stromelysin‐1 KO alone or combined with pretreatment of insulin, LA, and βME (Figure 4C). Single targeting stromelysin‐1 or together with insulin, LA, and βME achieved superior therapeutic efficacy against the monolayer hyperpermeability of IMECs exposed by high glucose.

### 3.5 Stromelysin‐1 KO Remodels the Secretome Towards a Pro‐Angiogenic Profile

**FIGURE 5:**
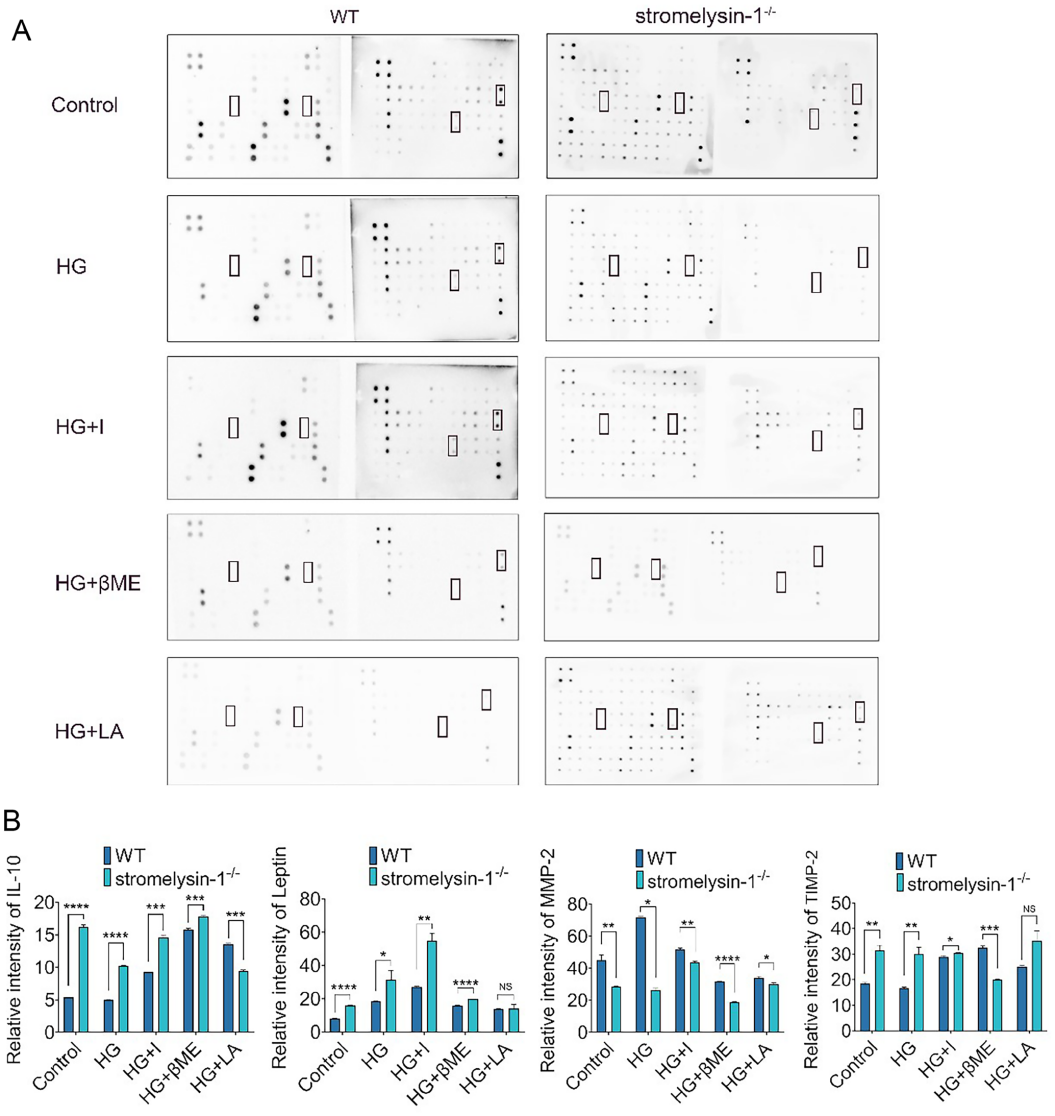
Stromelysin‐1 KO remodels the secretome of IMECs towards a pro‐stabilisation profile. (A) Representative cytokine array membranes comparing the secretomes of WT and stromelysin‐1 KO IMECs under 5.6 mM glucose (Control), 35 mM glucose (HG), 35 mM glucose plus 10^−8^ M insulin (HG + I), 35 mM glucose plus 0.5 mM l‐arginine (HG + LA) and 35 mM glucose plus 100 μM β‐mercaptoethanol (HG + βME). Black boxes highlight proteins with significant differential expression. Reference spots were used for signal normalisation. (B) Densitometric quantification of differentially secreted proteins. WT, wild‐type. KO, knockout. IMECs, islet microvascular endothelial cells. Data are expressed as mean ± SEM, *n* = 3. *p < 0.05, **p < 0.01, ***p < 0.001, ****p < 0.0001 compared with corresponding WT IMECs.

To elucidate the molecular mechanisms underlying the protective phenotypes observed upon stromelysin‐1 knockout, we profiled the secretome of both WT and stromelysin‐1 KO IMECs in the treatment medium using a cytokine antibody array. The analysis revealed that the genetic ablation of stromelysin‐1 led to significant alterations in the secretion of several key proteins implicated in microvascular homeostasis and inflammation (Figure 5A, Tables 1 and 2). Stromelysin‐1 KO resulted in a significant upregulation in the secretion of the anti‐inflammatory cytokine Interleukin‐10, the metabolic regulator and angiogenic factor leptin, and TIMP‐2. Conversely, the expression of MMP‐2, a key enzyme involved in extracellular matrix degradation and vascular remodelling, was significantly downregulated in stromelysin‐1‐deficient cells (Figure 5B). Supplementary treatment with insulin, lipoic acid, and β‐mercaptoethanol partially counteracted the effects of glucotoxicity, normalising the levels of these secreted factors towards those of the control groups. Collectively, these data suggest that the enhanced proliferation, migration, angiogenic capacity, and improved barrier integrity in stromelysin‐1 KO IMECs are, at least in part, mediated by a fundamental shift in their secretory profile, thereby creating a microenvironment conducive to endothelial cell function and resilience against glucotoxicity stress.

**TABLE 1:** Array map for cytokine arrays (left membrane).

	A	B	C	D	E	F	G	H	I	J	K	L	M	N
1	POS	POS	NEG	NEG	BLANK	Axl	BLC	CD30L	CD30	CD40	CRG‐2	CTACK	CXCL16	Eotaxin1
2	POS	POS	NEG	NEG	BLANK	Axl	BLC	CD30L	CD30	CD40	CRG‐2	CTACK	CXCL16	Eotaxin1
3	Eotaxin2	Fas Ligand	CX3CL1	GCSF	GM‐CSF	IFN gamma	IGFBP 3	IGFBP 5	IGFBP 6	IL‐1 alpha	IL‐1 beta	IL‐2	IL‐3	IL‐3 Rb
4	Eotaxin2	Fas Ligand	CX3CL1	GCSF	GM‐CSF	IFN gamma	IGFBP 3	IGFBP 5	IGFBP 6	IL‐1 alpha	IL‐1 beta	IL‐2	IL‐3	IL‐3 Rb
5	IL‐4	IL‐5	IL‐6	IL‐9	IL‐10	IL12 p40/70	IL‐12 p70	IL‐13	IL‐17	KC	Leptin R	Leptin	LIX	L Selectin
6	IL‐4	IL‐5	IL‐6	IL‐9	IL‐10	IL12 p40/70	IL‐12 p70	IL‐13	IL‐17	KC	Leptin R	Leptin	LIX	L Selectin
7	Ltn/XCL1	MCP‐1	MCP‐5	M‐CSF	MIG	MIP‐1 alpha	MIP‐1 gamma	MIP‐2	MIP‐3 beta	MIP‐3 alpha	PF‐4	P Selectin	RANTES	SCF
8	Ltn/XCL1	MCP‐1	MCP‐5	M‐CSF	MIG	MIP‐1 alpha	MIP‐1 gamma	MIP‐2	MIP‐3 beta	MIP‐3 alpha	PF‐4	P Selectin	RANTES	SCF
9	SDF‐1alpha	TARC	TCA‐3	TECK	TIMP‐1	TNF alpha	sTNFRI	sTNFRII	TPO	VCAM‐1	VEGF‐A	BLANK	BLANK	POS
10	SDF‐1alpha	TARC	TCA‐3	TECK	TIMP‐1	TNF alpha	sTNFRI	sTNFRII	TPO	VCAM‐1	VEGF‐A	BLANK	BLANK	POS

*Note:* Red colour represents significantly increased cytokines in stromelysin‐1 KO IMECs.

Abbreviation: IMECs, islet microvascular endothelial cells.

**TABLE 2:** Array map for cytokine arrays (right membrane).

	A	B	C	D	E	F	G	H	I	J	K	L
1	POS	POS	NEG	NEG	BLANK	bFGF	CD26	Dtk	E‐selectin	FcγRIIB	Flt‐3‐L	GITR
2	POS	POS	NEG	NEG	BLANK	bFGF	CD26	Dtk	E‐selectin	FcγRIIB	Flt‐3‐L	GITR
3	HGF‐R	ICAM‐1	IGFBP2	IGF‐1	IGF‐2	IL‐15	IL‐17BR	IL‐7	I‐TAC	Lungkine	MDC	MMP‐2
4	HGF‐R	ICAM‐1	IGFBP2	IGF‐1	IGF‐2	IL‐15	IL‐17BR	IL‐7	I‐TAC	Lungkine	MDC	MMP‐2
5	MMP‐3	OPN	OPG	Pro MMP‐9	Resistin	Shh‐N	TCK‐1	TIMP‐2	TRANCE	TROY	TSLP	VEGF R1
6	MMP‐3	OPN	OPG	Pro MMP‐9	Resistin	Shh‐N	TCK‐1	TIMP‐2	TRANCE	TROY	TSLP	VEGF R1
7	VEGFR2	VEGFR3	VEGFD	BLANK	BLANK	BLANK	BLANK	BLANK	BLANK	BLANK	BLANK	POS
8	VEGFR2	VEGFR3	VEGFD	BLANK	BLANK	BLANK	BLANK	BLANK	BLANK	BLANK	BLANK	POS

*Note:* Red colour represents significantly increased cytokines in stromelysin‐1 KO IMECs, green colour represents significantly decreased cytokines.

Abbreviation: IMECs, islet microvascular endothelial cells.

## 4 Discussion

The islets of Langerhans are highly vascularized mini‐organs, and preserving the structural integrity and physiological function of their microvascular endothelium is vital for endocrine function and protection against metabolic insults [19, 20]. As the primary cellular component of this microvasculature, IMECs are major targets of glucotoxicity‐induced injury [21], which fosters a pro‐thrombotic, pro‐inflammatory, and oxidant milieu. While this pathological cascade is well‐recognised, the specific molecular drivers remain incompletely defined. In the current study, we identify stromelysin‐1 (MMP‐3) as a mediator of the process, demonstrating that its genetic ablation attenuates glucotoxicity‐induced IMEC dysfunction by promoting a pro‐angiogenic and functionally stable endothelial phenotype.

Previous work has implicated gelatinolytic MMPs, specifically MMP‐2 and MMP‐9, in the dynamic remodelling of the ECM and the liberation of pro‐inflammatory mediators. However, the roles of other MMPs, particularly the upstream activators, have remained less clear. Stromelysin‐1 is a key member of the MMP family with broad substrate specificity [22, 23], capable of degrading fibronectin, laminin, elastin, and various collagens [24, 25, 26, 27]. Stromelysin‐1 functions as a potent upstream activator for other pro‐MMPs, including pro‐MMP‐2 and pro‐MMP‐9. Therefore, its expression and activation can initiate a proteolytic cascade that drives extensive tissue remodelling. Our finding that stromelysin‐1 knockout leads to a significant downregulation of MMP‐2 protein expression provides evidence for this hierarchical activation cascade operating within IMECs. This line of evidence establishes stromelysin‐1 as a pivotal initiator of the ECM degradation cascade under glucotoxicity stress. Considering the ECM's dual role as a structural scaffold and a repository for signalling molecules, its degradation by stromelysin‐1 inevitably leads to multi‐faceted impacts on IMEC fate.

Microvascular endothelial cell function is fundamentally determined by a balance of proliferation, migration, capillary‐like tube formation, and monolayer permeability. Our data demonstrate that stromelysin‐1 acts as a negative regulator across the spectrum of functions. The enhanced proliferation and migration observed in stromelysin‐1 KO cells are foundational for neovascularization. The processes culminated in a coordinated increase in tube formation, suggesting that stromelysin‐1 knockout orchestrates a functional pro‐angiogenic program, rather than just stimulating isolated cellular behaviours.

Perhaps the most clinically relevant finding is the role of stromelysin‐1 in regulating endothelial barrier integrity. A hallmark of diabetic microangiopathy is the breakdown of the endothelial barrier, leading to hyperpermeability [28]. Stromelysin‐1 is known to directly attack basal lamina components and tight junction proteins that are essential for this barrier [29]. Our study confirms the mechanism in IMECs, as stromelysin‐1 depletion restored monolayer permeability to near‐physiological levels even under high‐glucose challenge. The protective effects of stromelysin‐1 knockout on IMEC function were phenocopied by treatment with antioxidants lipoic acid and βME [30]. The phenotypic convergence reveals a hierarchical organisation in the pathogenesis of glucotoxic endothelial injury, firmly positioning stromelysin‐1 as a primary downstream executioner of oxidative stress. In diabetic microvascular complications, ROS are potent signalling molecules that drive MMP‐3 expression via redox‐sensitive transcription factors such as NF‐κB and AP‐1 [31, 32]. Furthermore, ROS can directly activate latent pro‐MMPs through the oxidation of the autoinhibitory pro‐domain through the cysteine switch mechanism [33]. Therefore, while βME neutralises the upstream oxidative trigger, stromelysin‐1 ablation severs the downstream structural degradation pathway, yielding a comparable preservation of endothelial integrity. The paradigm also contextualises the vasoprotective efficacy of insulin and l‐arginine observed in our study. Glucotoxicity‐induced ROS rapidly depletes NO bioavailability via eNOS uncoupling. While l‐arginine bypasses this metabolic bottleneck and insulin drives the PI3K/Akt/eNOS axis [34], their sustained vasoprotective efficacy fundamentally relies on an intact extracellular matrix and glycocalyx to facilitate mechanotransduction and receptor scaffolding. By preventing ROS‐mediated matrix proteolysis, stromelysin‐1 depletion likely maintains the essential structural microenvironment required for insulin and NO signalling to function optimally. Together, these findings support a model wherein oxidative stress initiates the glucotoxic cascade, but aberrant stromelysin‐1 activity acts as the definitive structural executioner driving islet endothelial dysfunction.

To elucidate the underlying molecular basis for these functional improvements, we investigated the secretome of the IMECs. Vascular inflammation and angiogenesis are tightly regulated by a network of secreted factors [35, 36]. Our cytokine array analysis revealed that stromelysin‐1 deficiency fundamentally remodels the IMEC secretome, shifting it from a pro‐inflammatory, matrix‐degrading state towards an anti‐inflammatory and pro‐stabilisation profile. Stromelysin‐1 ablation does not reduce downstream MMP‐2 expression solely; rather, it re‐establishes the stoichiometric balance between MMP‐2 and TIMP‐2. Reconstitution of proteolytic equilibrium prevents excessive extracellular matrix degradation and preserves the integrity of the microvascular niche. Maintenance of the basal lamina, in turn, sustains the mechanotransductive architecture required for endothelial cell survival, junctional stability, and physiological capillary morphogenesis [37]. Concurrently, the upregulation of the potent anti‐inflammatory cytokine IL‐10 can explain the dampened inflammatory state [38], as IL‐10 is known to protect endothelial function by inhibiting pro‐inflammatory cytokine production and attenuating superoxide generation [39]. Furthermore, the increase in leptin, a factor known to enhance eNOS expression and nitric oxide production, likely contributes to the observed improvements in endothelial homeostasis and angiogenic capacity [40]. Therefore, stromelysin‐1 appears to orchestrate a dual assault on IMECs: direct proteolytic damage to the ECM and cell junctions, and indirect damage by fostering a pro‐inflammatory and matrix‐degrading secretome.

While our study investigates the role of the stromelysin‐1 pathway in glucotoxicity‐induced endothelial dysfunction, certain limitations should be noted. The current experiments rely on the immortalised MS1 cell line. Although MS1 cells retain basic endothelial characteristics, they may exhibit altered metabolic responses compared to primary cells and lack the cellular crosstalk present in the intact islet microenvironment. Therefore, future studies are needed to validate these findings in primary mouse or human islet microvascular endothelial cells. Additionally, in vivo investigations using diabetic animal models, such as those employing endothelial‐specific stromelysin‐1 knockout or targeted pharmacological inhibition, are required to determine the physiological relevance of this pathway in maintaining islet vascular integrity.

In conclusion, our research establishes stromelysin‐1 as a key regulator of IMEC homeostasis under glucotoxicity. Its ablation blocks a single pathological event but triggers a multi‐faceted protective response and a systemic shift towards a pro‐angiogenic secretome. MMP‐3 knockout could be a therapeutic target for preserving islet microvascular function in diabetes.

## Author Contributions

Conceptualization: Bing Wang, Mingming Liu, Ruijuan Xiu; Methodology: Bing Wang, Mingming Liu, Yuan Li; Investigation: Bing Wang, Weiqi Liu, Yuan Li, Qin Ouyang, Yingyu Wang, Xiang Xu, Bingwei Li, Xu Zhang; Formal analysis and investigation: Bing Wang, Weiqi Liu, Yuan Li, Qin Ouyang, Yingyu Wang, Xiang Xu, Bingwei Li, Xu Zhang; Writing ‒ original draft preparation: Bing Wang, Weiqi Liu; Writing ‒ review and editing: Mingming Liu; Funding acquisition: Mingming Liu; Supervision: Mingming Liu.

## Funding

This study was financially supported by grants from the Beijing Municipal Natural Science Foundation (7252093) and the National Natural Science Foundation of China (81900747).

## Conflicts of Interest

The authors declare no conflicts of interest.

## Data Availability

The data that support the findings of this study are available from the corresponding author upon reasonable request.
